# Factors Influencing SVF Yields from Human Adipose Tissue: Isolation Technique, Age, and Sex

**DOI:** 10.3390/jcm15052051

**Published:** 2026-03-08

**Authors:** Sarah Regener, Elijah Joy, Kristin Comella, Sunny Kim

**Affiliations:** Progressive Rehabilitation Medicine, 6005 Rockwell Dr. NE, Cedar Rapids, IA 52403, USA; sarah.regener@prmpractice.com (S.R.); ejoy@student.touro.edu (E.J.); kristin.comella@prmpractice.com (K.C.)

**Keywords:** stromal vascular fraction, adipose-derived stem cells, mesenchymal stem cells, mechanical isolation, enzymatic isolation, regeneration, percent viability, stem cell count

## Abstract

**Background/Objectives:** Stromal vascular fraction (SVF) from adipose tissue contains regenerative cell populations, including adipose-derived stem cells (ADSCs), and is increasingly used in clinical therapies. However, the effects of isolation technique and donor characteristics on SVF yield and viability remain unclear. This study aims to assess the impact of mechanical versus enzymatic isolation, as well as donor age and sex, on SVF total nucleated cell count (TNC) and viability. **Methods:** A retrospective analysis was conducted on 114 patients undergoing ADSC harvesting via a mini-liposuction. SVF was isolated using enzymatic digestion (*n* = 100) or mechanical digestion (*n* = 14). Percent viability and TNC were assessed using the Chemometec NC-200 NucleoCounter^®^. The influence of isolation technique, donor age, and donor sex on SVF yield and viability was evaluated using Pearson’s correlation and independent *t*-tests. **Results:** Enzymatic digestion yielded significantly higher cell viability compared to mechanical isolation (*p* < 0.001), although no significant difference in TNC was observed between the two methods. Increasing donor age was modestly associated with reduced viability in enzymatically processed samples but not in mechanically processed samples. Donor age showed no significant association with TNC for either isolation method. Donor sex was not correlated with viability in either group; however, female donors exhibited significantly higher TNC following enzymatic digestion, a trend not observed with mechanical isolation. **Conclusions:** Enzymatic digestion preserves cell viability more effectively than mechanical methods, while donor age and sex have variable effects depending on the isolation protocol. These findings underscore the importance of considering both biological and methodological factors in SVF preparation for clinical use. Further studies with larger, balanced cohorts are needed to validate these results.

## 1. Introduction

### 1.1. Adipose-Derived Stem Cells

Mesenchymal stem cells (MSCs) have generated significant interest in regenerative medicine due to their multipotent capacity to differentiate into multiple tissue lineages, including chondroblasts, osteoblasts, and fibroblasts. In addition to their multipotency, MSCs contribute to tissue repair by creating a regenerative microenvironment at the local site of injury. This occurs primarily through paracrine signaling, which involves the release of cytokines, growth factors, secretomes, and chemokines that modulate inflammation, promote cellular recruitment, and support tissue regeneration [1,2]. Through these combined regenerative mechanisms, MSCs have demonstrated efficacy in treating a wide variety of conditions, including cardiovascular disease, liver disease, spinal cord injury, orthopedic injuries, and autoimmune diseases [3,4].

Adipose tissue has been identified as a reliable, cost-effective, and minimally invasive source of MSCs [5]. When compared to other sources of MSCs, such as bone marrow, adipose tissue has a higher concentration of stem cells and supports a greater rate of stem cell proliferation [6]. Stem cells isolated from adipose tissue are referred to as adipose-derived stem cells (ADSCs). ADSCs display hallmark MSC properties, such as a fibroblast-like morphology, multipotent differentiation capacity, and expression of characteristic MSC surface markers [7].

ADSCs offer several advantages over other types of stem cells. While bone marrow-derived mesenchymal stem cells (BMSCs) are well recognized for their strong osteogenic and chondrogenic differentiation potential [8], extraction of BMSCs can be painful, invasive, costly, and associated with an increased risk of adverse events [9,10]. In contrast, ADSCs can be readily obtained in large quantities through a mini-liposuction procedure, offering an economical and minimally invasive alternative [11].

In addition to the ease of harvesting, ADSCs also exhibit robust regenerative potential. ADSCs have demonstrated a greater proliferative capacity and survive longer in culture compared to BMSCs [12,13]. Like other types of MSCs, much of the therapeutic ability of ADSCs is mediated via the release of paracrine factors. Through the secretion of various cytokines and growth factors, ADSCs have been shown to contribute to angiogenesis [14], immunomodulation [15], and neuroprotection [16]. As such, ADSCs have demonstrated therapeutic efficacy in a wide variety of conditions, including osteoarthritis [17,18,19], wound healing [20], and neurologic disorders [21,22,23].

Through a mini-liposuction procedure, adipose tissue can be easily harvested in a clinical setting. Following collection, mature adipocytes are removed to isolate the stromal vascular fraction (SVF). SVF is a heterogeneous mixture of cells containing ADSCs, pericytes, smooth muscle cells, macrophages, lymphocytes, endothelial progenitor cells, and preadipocytes [24]. SVF has been observed to induce angiogenesis [25], mediate the immune response [26], regenerate various tissue types [27], and contribute to cellular adhesion and matrix remodeling [28].

In recent decades, SVF has emerged as a clinically significant intervention in regenerative medicine due to its ability to provide therapeutic benefits without the need for ex vivo cell expansion; SVF can be isolated, prepared, and administered within the same clinical setting [29,30]. Consequently, the safety and efficacy of SVF as a therapeutic strategy have been extensively studied. Autologous SVF is associated with favorable safety profiles and low rates of serious adverse events [31,32,33]. Moreover, accumulating evidence supports its therapeutic benefits across a range of pathological conditions characterized by inflammation, ischemia, and impaired tissue repair [34,35,36]. Reported clinical outcomes include reduction in pain and increased functional capacity [32,36]. Collectively, these findings underscore the growing recognition of SVF as a practical and clinically effective regenerative modality.

### 1.2. Isolation Technique

Isolation of the SVF from the lipoaspirate can be achieved through either enzymatic or mechanical processing methods. Enzymatic digestion is commonly achieved via the use of collagenase, a proteolytic enzyme that degrades the collagenous extracellular matrix (ECM) that binds adipocytes together [37]. Conversely, mechanical digestion relies on physical force or filtration to break apart the adipose tissue without enzymatic aid. Mechanical methods are often praised for being simpler, faster, cheaper, and less burdened by regulatory issues related to “manipulation”, whereas enzymatic digestion maintains advantages such as an enhanced efficiency in breaking down the ECM [38].

To date, the question of which method is superior remains unresolved. Attempts have been made to compare the two methods, but the lack of a standardized protocol has limited the ability to draw direct conclusions [39,40]. Additionally, conflicting reports exist regarding which method yields a higher cell count (defined as the total number of nucleated cells present in a sample of SVF) and a greater preservation of cell viability (defined as the proportion of nucleated cells still alive in a sample of SVF). Senesi et al. (2019) found that enzymatic digestion resulted in a significantly higher cell viability compared to mechanical processing [41]. Other studies have contrasted this finding, noting that while enzymatic digestion produced a much higher cell count, there was no significant difference in cell viability between the two methods [42]. Solodeev et al. similarly reported that enzymatic isolation yielded nearly twice the number of nucleated SVF cells as mechanical isolation, but maintained that the resulting mechanical yields were comparable to enzymatic yields reported in various other clinical trials [43]. More recently, a 2024 systematic review article by Uguten et al. determined that enzymatic and mechanical digestion produced comparable results for both cell yield and cell viability across 33 different isolation procedures [44]. Collectively, these varying findings highlight the need for further investigation to clarify the impact of processing technique on SVF yield and viability.

### 1.3. Age

In addition to methodological differences, donor characteristics such as age may also impact SVF yield and viability. Given that overall stem cell counts decline with age [45], concerns have been raised that harvesting and re-implanting ADSCs may be less effective in elderly patients than in younger ones. To compound this concern, ADSCs have been shown to exhibit age-related senescent changes, including a loss of proliferation capacity and decreased osteogenic differentiation potential [46,47]. Age may also impact the paracrine activity of ADSCs, as ADSCs obtained from older donors show impaired secretion of cytokines and growth factors [48].

A variety of mechanisms have been proposed to explain how stem cells age. One theory attributes cellular senescence to telomere attrition, which cells may interpret as DNA damage and consequently activate stress pathways that inhibit proliferation [49]. However, MSC telomere lengths have been shown to remain relatively stable as humans age, indicating that stem cells may possess a built-in mechanism to protect their telomeres from age-related shortening [50]. An alternate explanation could involve DNA damage resulting from oxidative stress, leading to premature cell-cycle arrest and dysfunctional stem cell activity [51]. Another potential contributing factor could be inflammatory changes in the microenvironment of MSCs, which may induce senescent behavior, such as excess release of pro-inflammatory cytokines and reduced proliferative capacity [52].

Despite these functional declines, the relationship between age and SVF yield remains inconclusive. While some studies report a negative correlation between donor age and SVF yield [53,54], others have found no significant difference in SVF yields between younger and older individuals [55,56,57]. The relationship between donor age and SVF viability is even less defined. Although some evidence suggests that ADSCs from older donors may demonstrate reduced viability [48], most studies focus on cell yield rather than directly evaluating age-related differences in cell viability. This leaves a significant gap in research regarding the true impact of donor age on SVF yield and viability.

### 1.4. Sex

An additional factor with the potential to influence SVF yield and viability is donor sex. Studies have shown that 17-β estradiol (E2) can positively impact the proliferation and adipogenic differentiation capacity of ADSCs in vitro [58]. This effect may be due to E2’s ability to activate important cell-survival and pro-growth signaling pathways such as PI3K/AKT and MAPK, a process that has been demonstrated in both human bone marrow MSCs [59] and cochlear mouse cells [60]. E2 may also promote the secretion of growth factors such as vascular endothelial growth factor (VEGF), leading to increased cellular vascularization and greater rates of cell survival [58]. Given that ADSCs have been shown to express estrogen receptors type α and β (ERα and ERβ) [61], it is plausible that differences in donor sex hormones could contribute to variability in SVF outcomes.

However, this relationship remains unclear, as previous attempts to quantify a potential correlation report inconsistent findings. Collon et al. (2022) found that women yielded a significantly higher cell count than men [62]. Similarly, Sari et al. (2022) reported greater cell yields in women, but found that male donors exhibited higher cell viability [63]. In contrast, Andjelkov et al. (2023) found no statistically significant difference between men and women for either cell yield or viability [64]. These contradictory findings showcase the need for further research to elucidate the true effect of donor sex on SVF outcomes.

To evaluate the effects of varying isolation techniques and patient factors on SVF viability and yield, we conducted a retrospective analysis of 114 patient charts from individuals who underwent an ADSC harvesting procedure at our clinic from 19 January 2023 to 11 December 2025. We hypothesize that enzymatic isolation will outperform mechanical isolation in both yield and viability. SVF yield and viability will decrease as age increases, and female donors will demonstrate higher SVF yield and viability compared to male donors.

## 2. Materials and Methods

### 2.1. Study Design

This study followed a retrospective observational design aimed at comparing the influence of patient factors and isolation technique on SVF outcomes. Both isolation methods were part of routine clinical practice during the study period. Mechanical isolation using the Q-graft^®^ system (REF 300000, Human Med AG, Schwerin, Germany) was utilized earlier, after which the clinic transitioned to enzymatic isolation using the Time Machine 3.0 Automated Cell System (ACS Combo-B Incubator, Model ACS-102, BSL Co., Ltd., Busan, Republic of Korea). Quantitative SVF analysis using the NucleoCounter^®^ (Product No. 900-0201, Chemometec A/S, Allerød, Denmark) did not become a routine part of clinical procedure until shortly before this transition. As a result, fewer mechanical cases were available for quantitative analysis, reflecting an evolution in procedural standards rather than patient-specific selection criteria.

### 2.2. Manual Pull Harvesting

A total of 100 patients underwent a mini-liposuction procedure to the lower abdominal subcutaneous region using a manual pull technique. All procedures were performed by either a licensed physician or nurse practitioner. Before the procedure, Acoustic Pressure Wave Therapy was administered to the abdominal area using the Miracle Wave HP50 (Model Hi-Puls HP50, W-Medical, Namyangju-si, Gyeonggi-do, Republic of Korea) to reduce pain and swelling and promote tissue healing. Once transferred to the procedure room, patients were positioned supine and monitored non-invasively for continuous pulse oximetry, heart rate, and demand blood pressure. PRONOX was used for pain and analgesia throughout the procedure.

The abdomen was sterilely prepped with Hibiclens, and a sterile field was laid. A tumescent solution containing 200 mL of normal saline, 25 mL of 1% lidocaine, 5 mL of 8.4% sodium bicarbonate, and 0.5 mL of epinephrine (1:1000) was prepared in a sterile fashion before the procedure. The left lower abdomen was injected with 2 mL of the tumescent solution and penetrated with a #11 blade to make a small stab incision. The incision was then infiltrated with a sterile 1.6 mm/15 cm infiltration cannula. Using a slow manual pull technique, a total volume of 250 mL of tumescent solution was administered to anesthetize the lower abdominal subcutaneous fat. Care was taken to evenly distribute the solution and avoid damage to muscles, nerves, great vessels, and barotrauma. Then, a 2.4 mm/15 cm cannula was used to perform a manual pull lipoaspiration on the anesthetized region. The incision site was closed with sterile strips and covered with an ABD pad. Lastly, an abdominal binder was used postoperatively for compression.

### 2.3. Enzymatic Isolation of the SVF (Post Manual Pull Harvesting)

A total of 100 mL of adipose tissue was extracted via a sterile manual pull lipoaspiration technique.

Half of the lipoaspirate was packed on ice and shipped to American Cell Technology for banking and culturing. The remaining 50 mL were then processed for SVF isolation.

The fat was first centrifuged using the Time Machine 3.0 Automated Cell System (ACS Combo-B Incubator, Model ACS-102, BSL Co., Ltd., Busan, Republic of Korea) at 2800 RPM for 3 min to separate adipocytes from the tumescent solution. The tumescent layer was discarded, and 3 mL of CSN-TMAX^®^ collagenase (21.5 Wünsch Units, GMP grade; Cell Surgical Network Inc.^®^, Time Machine Accelerator, Rancho Mirage, CA, USA) suspended in 25 mL of normal saline was added to the isolated adipocytes. The adipose-collagenase mixture was then incubated at 36 °C for 45 min.

Following incubation, the mixture was transferred into an Automatic Tissue Processing Unit (ATPU) (Fat Concentration System, Model ACPU-100, BSL Co., Ltd., Busan, Republic of Korea) along with 150 mL of 5% dextrose in lactated Ringer’s (D5LR). The ATPU was then placed into the Time Machine 3.0, where it underwent three automated cycles of centrifugation and washing with D5LR. Each washing cycle lasted approximately 8 min. The centrifugation speeds and flow parameters varied throughout the cycle according to manufacturer-programmed settings. Approximately 10 mL of purified SVF was then extracted, passed through a 100 µm filter (Adinizer, REF SKT-An-101, BSL Co., Ltd., Busan, Republic of Korea), and prepared for immediate use.

### 2.4. Water-Assisted Harvesting

A total of 14 patients underwent a water-assisted mini-liposuction procedure of the lower abdominal subcutaneous region. All procedures were performed by either a licensed physician or nurse practitioner. Patients were processed as described in Section 2.1, with preoperative Acoustic Pressure Wave Therapy and continuous intraoperative monitoring of vital signs. PRONOX was administered for pain and analgesia.

The abdomen was sterilely prepped with betadine, and a sterile field was laid. A tumescent solution containing 500 mL of normal saline, 25 mL of 2% lidocaine, 5 mL of 8.4% sodium bicarbonate, and 1 mL of epinephrine (1:1000) was prepared in a sterile fashion before the procedure. The left lower abdomen was injected with 3 mL of tumescent solution and penetrated with a #11 blade to make a small stab incision. The incision was then infiltrated with a sterile 2.5 mm/15 cm infiltration cannula. Using the body-jet^®^ evo water-assisted liposuction device (REF 500000-17, Human Med AG, Schwerin, Germany), a total volume of 500 mL of tumescent solution was administered to anesthetize the lower abdominal subcutaneous fat. Care was taken to evenly distribute the solution and avoid damage to muscles, nerves, great vessels, and barotrauma. Then, a 3.5 mm/15 cm cannula was used to perform a water-assisted lipoaspiration on the anesthetized region using the body-jet^®^ evo (REF 500000-17, Human Med AG, Schwerin, Germany). The incision site was closed with sterile strips and covered with an ABD pad. Lastly, an abdominal binder was used postoperatively for compression.

### 2.5. Mechanical Isolation of the SVF (Post Water-Assisted Harvesting)

The Q-graft^®^ collector (REF 300000, Human Med AG, Schwerin, Germany) was used for isolation of the SVF according to the manufacturer’s automated processing protocol. All separation and filtration steps were performed within the closed system and were not user-adjustable. As the adipose tissue was being harvested, the lipoaspirate was funneled directly into the upper chamber of the Q-graft^®^ collector. A total of 75 mL of adipose tissue was collected. Waste products were removed automatically. Upon completion of the lipoaspiration, the harvested lipoaspirate was incubated at 38 °C for 45 min. No enzymes were used during this process. Immediately following incubation, fractional cell separation and cross-flow filtration were performed to mechanically isolate the SVF. Approximately 20 mL of SVF was then extracted, passed through a 100 µm filter (Adinizer, REF SKT-AN-101, BSL Co., Ltd., Busan, Republic of Korea), and prepared for immediate use [65].

### 2.6. NucleoCounter^®^

Following both mechanical and enzymatic isolation protocols, percent viability and total nucleated count (TNC) were determined for the entire SVF isolate. To start, 0.2 mL of each isolate was loaded into a Via1 Cassette^TM^ (Product No. 941-0011, ChemoMetec A/S, Allerød, Denmark) and inserted into the NucleoCounter^®^ NC-200^TM^ (Product No. 900-0201, Chemometec A/S, Allerød, Denmark) for analysis. The NucleoCounter^®^ utilizes two fluorescent stains: acridine orange (AO) and 4′,6-diamidino-2-phenylindole (DAPI). AO binds to the nucleic acids of all nucleated cells present in the sample, quantifying the total number of nucleated cells present per milliliter of SVF. This quantity is then multiplied by the total volume of SVF obtained to determine the TNC of the entire sample. In contrast, DAPI is cell-impermeable and selectively stains only the nucleic material of dead cells, revealing the proportion of the TNC that is still alive (percent viability).

### 2.7. Statistical Analysis

To analyze the effect of the isolation technique and a variety of patient factors on percent viability and TNC, a retrospective review of 114 patient charts was conducted. The patients were grouped into two categories based on isolation protocol: Time Machine 3.0 (*n* = 100) and Q-graft^®^ (*n* = 14). Python 3.13.7 was used to generate graphs depicting the relationship between isolation technique, patient-specific variables, percent viability, and TNC. Pearson’s Correlation coefficient was used to evaluate the relationship between continuous variables, and an independent samples *t*-test was used to evaluate groups defined by categorical variables. Statistical significance was set at *p* < 0.05. A single asterisk (*) was used to denote *p*-values less than 0.05, a double asterisk (**) was used to denote *p*-values less than 0.01, and a triple asterisk (***) was used to denote *p*-values less than 0.001. All *p*-values greater than 0.05 were denoted with ns (non-significant).

In addition, given the uneven sample sizes between the two isolation methods, a post hoc power analysis was performed for the primary comparison between isolation techniques using Cohen’s *d* and a two-tailed independent samples *t*-test (α = 0.05).

Lastly, considering that the age distribution for the mechanical isolation group (50–88 years) is older than that of the enzymatic isolation group (21 to 88 years), a sensitivity analysis was performed using linear regression models to evaluate whether associations between isolation technique and SVF outcomes remained after adjusting for donor age.

## 3. Results

### 3.1. Demographics and Corresponding Means

Table 1 summarizes the distribution of patient characteristics, along with corresponding mean cell yield and mean cell viability observed in SVF isolates. The study included 114 patients, with an equal number of males and females (57 each, 50% respectively). Females demonstrated both a higher mean cell yield and mean cell viability than men. Patients were grouped into 3 categories based on age: 20–40 years old (*n* = 7, 6.1%), 40–60 years old (*n* = 34, 29.8%), and 60–90 years old (*n* = 73, 64.0%). The highest mean yield was observed in the 40–60 year group (7.05 × 10^6^), followed by the 60–90 year group (5.73 × 10^6^), and finally the 20–40 year group (5.39 × 10^6^). Sex distribution within age categories was not perfectly balanced; females comprised approximately 42.9%, 40.6%, and 54.7% of the 20–40, 40–60, and 60–90 groups, respectively, indicating a slight male predominance in younger groups and a slight female predominance in older groups. The highest mean viability was observed in the youngest group (85.94%), followed by the 40–60 year group (83.01%), and finally the 60–90 year old group (81.62%). Most patients underwent enzymatic isolation (*n* = 100, 87.7%), with only 14 patients undergoing mechanical digestion (12.3%). Enzymatic digestion resulted in a higher mean yield and viability (6.48 × 10^6^, 85.8% respectively) than mechanical digestion (3.31 × 10^6^, 57.1% respectively).

### 3.2. Isolation Technique

**Figure 1:**
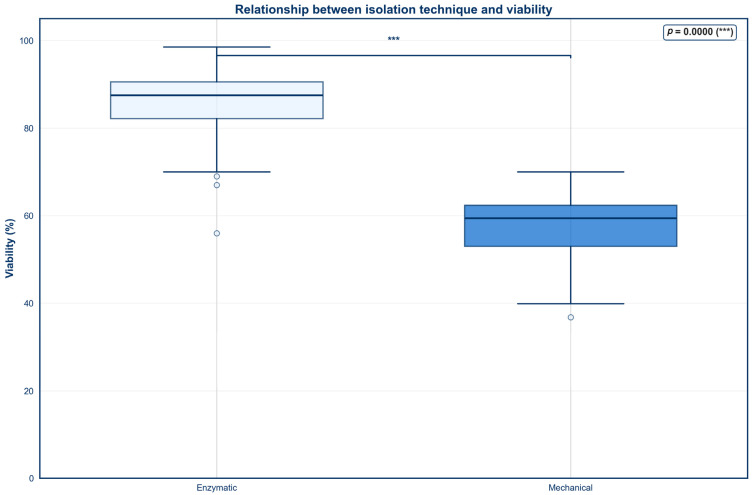
Relationship between isolation technique and viability.

**Figure 2:**
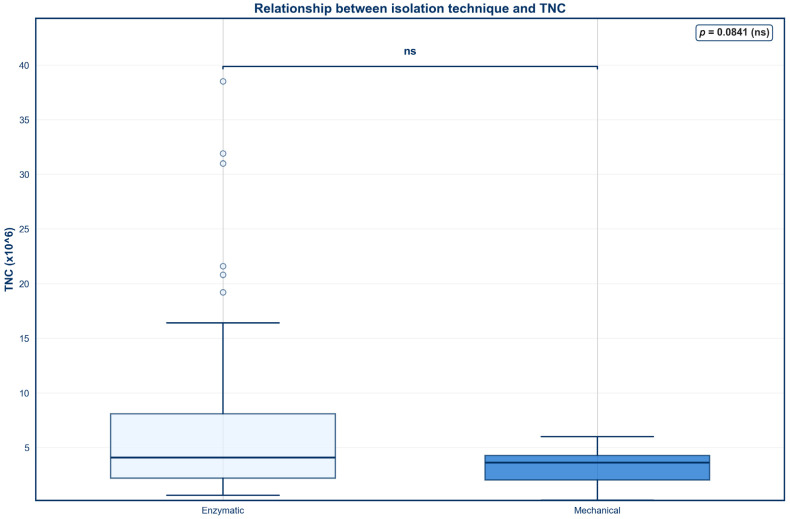
Relationship between isolation technique and TNC.

A box and whiskers plot was created to evaluate the association between isolation technique and percent viability (Figure 1). Enzymatic digestion resulted in a significantly higher median viability when compared to mechanical digestion (*p* < 0.001). Both the upper and lower quartiles were shifted upward for enzymatic digestion, indicating a consistently higher percent viability across the majority of the samples. However, no statistically significant difference in TNC was found between the two isolation techniques (Figure 2; *p* > 0.05).

To account for the unequal sample sizes between enzymatic digestion (*n* = 100) and mechanical digestion (*n* = 14), a post hoc power analysis was conducted using Cohen’s *d* and a two-tailed independent samples *t*-test (α = 0.05). For percent viability, post hoc power analysis revealed a very large effect size (Cohen’s *d* = 3.68), resulting in a statistical power greater than 0.99 despite unequal group sizes. In contrast, the post hoc power analysis for TNC demonstrated a moderate effect size (Cohen’s *d* = 0.50), resulting in a statistical power of 0.41.

Because the age distribution differed between cohorts, with the enzymatic digestion group including donors ranging from 21 to 88 years and the mechanical isolation group including donors ranging from 50 to 88 years, a sensitivity analysis was conducted using linear regression models including isolation technique and donor age. After adjustment, enzymatic isolation remained significantly associated with higher percent viability (β = 28.5, *p* < 0.001), while donor age was not significantly associated with viability (*p* = 0.125). Additionally, isolation technique was not significantly associated with TNC after age adjustment (*p* = 0.091), and donor age was also not associated with TNC (*p* = 0.583).

### 3.3. Age

**Figure 3:**
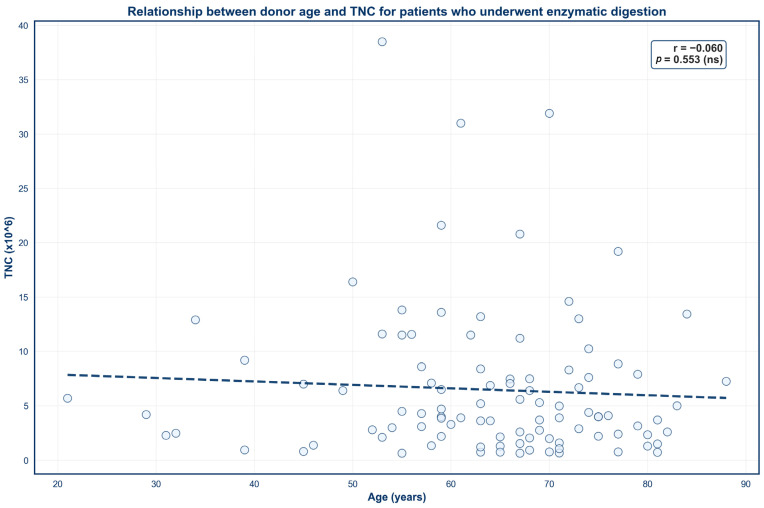
Relationship between donor age and TNC for patients who underwent enzymatic digestion. Circles represent TNC values from individual donors and dashes represent the linear regression line.

**Figure 4:**
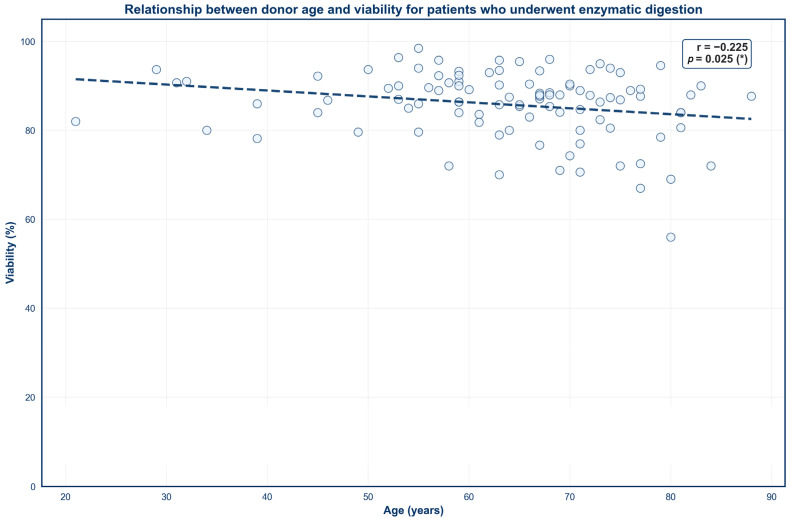
Relationship between donor age and viability for patients who underwent enzymatic digestion. Circles represent percent viability values from individual donors and dashes represent the linear regression line.

**Figure 5:**
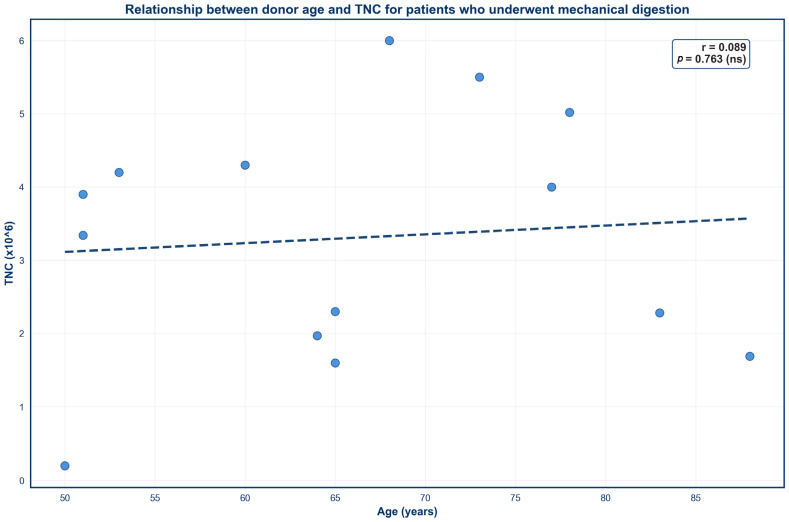
Relationship between donor age and TNC for patients who underwent mechanical digestion. Circles represent TNC values from individual donors and dashes represent the linear regression line.

**Figure 6:**
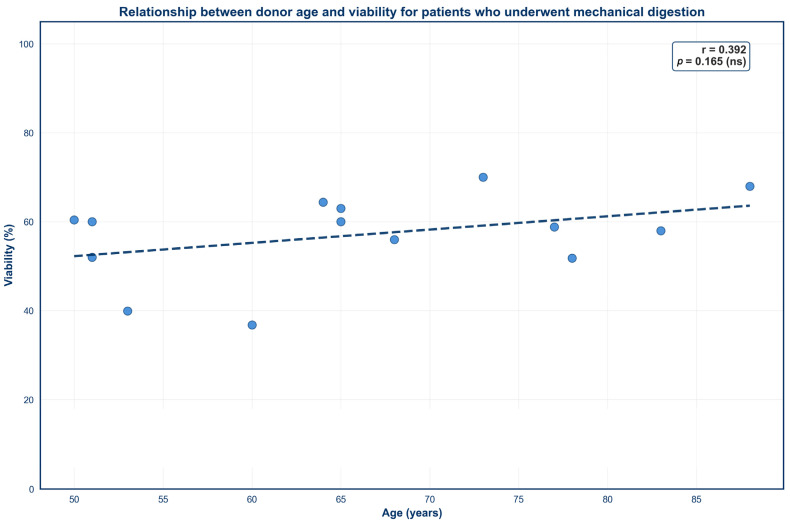
Relationship between donor age and viability for patients who underwent mechanical digestion. Circles represent percent viability values from individual donors and dashes represent the linear regression line.

Four scatter plots were created to demonstrate the impact of donor age on SVF outcomes across both isolation techniques (Figure 3, Figure 4, Figure 5 and Figure 6). No significant correlation was observed between donor age and TNC for either mechanical or enzymatic digestion (Figure 3 and Figure 5; *p* > 0.05). In contrast, donor age was negatively correlated with SVF viability for samples that were processed enzymatically (Figure 4; *p* < 0.05). This finding was not mirrored in mechanically isolated samples, with no significant correlation identified between donor age and SVF viability (Figure 6; *p* > 0.05).

### 3.4. Sex

**Figure 7:**
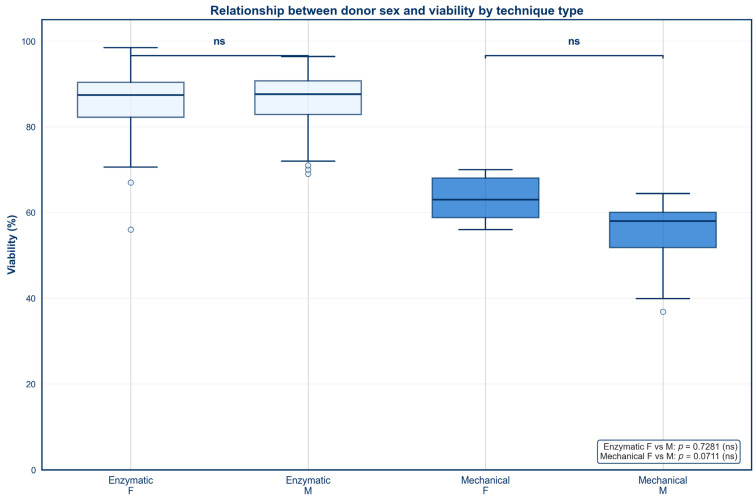
Relationship between donor sex and viability by technique type.

**Figure 8:**
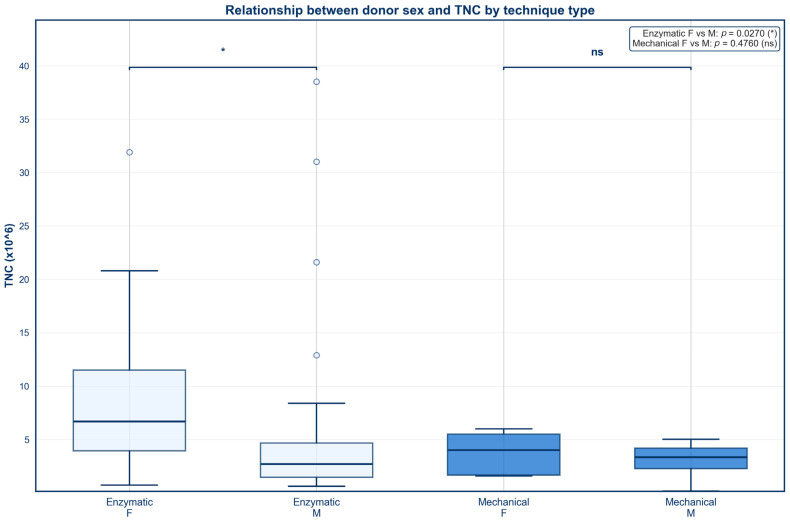
Relationship between donor sex and TNC by technique type.

Box and whisker plots were created to identify the impact of donor sex on SVF outcomes across both mechanical and enzymatic isolation methods (Figure 7 and Figure 8). No statistically significant difference in percent viability was found between males and females for either isolation type (Figure 7, *p* > 0.05). In contrast, females who underwent enzymatic digestion produced a significantly higher TNC than males (Figure 8, *p* < 0.05). No statistically significant difference in TNC was observed between males and females who underwent mechanical isolation (Figure 8, *p* > 0.05).

## 4. Discussion

SVF has been widely studied for its ability to promote tissue repair, regulate the immune system, and suppress inflammation [66]. SVF has demonstrated therapeutic efficacy in a broad range of clinical applications, such as treatment for osteoarthritis [67], wound healing [68], and ischemic heart failure [34]. To better understand factors influencing SVF characteristics, this study retrospectively evaluated the impact of isolation technique, donor age, and donor sex on SVF yield and viability in a clinical cohort of 114 patients who underwent ADSC harvesting via a mini-liposuction. To our knowledge, this study represents the first study to analyze the impact of various donor factors on SVF yield and viability across multiple isolation protocols.

### 4.1. Isolation Technique

As expected, enzymatic digestion was positively associated with higher percent viabilities. This result is likely attributed to the ability of proteolytic enzymes to gently break down the ECM of adipose tissue. By chemically loosening this structural network, stromal cells can be released with minimal physical stress, resulting in a greater preservation of cellular integrity [38]. In contrast, mechanical digestion relies on physical disruption of the lipoaspirate, which fails to fully degrade the ECM and subjects the cells to increased trauma during the isolation process. This can contribute to cellular damage and reduced viability [69]. This finding is supported by previous studies, which have also demonstrated that enzymatic isolation is more effective in preserving cell viability [38,41].

Contrary to our hypothesis, no significant correlation was found between isolation technique and TNC. This suggests that while enzymatic digestion improves the proportion of viable cells, it does not necessarily increase the total number of nucleated cells recovered. This result contrasts with prior studies, which have reported that enzymatic digestion yields higher TNCs [42]. However, this finding should be cautiously interpreted, as unequal sample sizes across the two isolation techniques may have reduced the ability to detect moderate differences, as reflected by the limited statistical power (power = 0.41) observed for this comparison.

Another potential confounding variable to consider is the uneven age distribution across the two isolation protocols. To account for this, a sensitivity analysis adjusting for donor age using a linear regression model was conducted, revealing that enzymatic isolation remained strongly associated with higher percent viability, while no association between isolation technique and TNC was observed after adjustment. These findings suggest that the observed differences in viability are unlikely to be explained by age differences between cohorts and can instead be attributed to isolation technique.

Lastly, the age groups were not perfectly balanced by sex. However, the differences were modest and unlikely to account for the observed age-related trends, although a minor contribution of sex distribution cannot be completely discounted.

### 4.2. Age

Donor age demonstrated a weak negative correlation with percent viability for SVF samples subjected to enzymatic digestion, whereas no such relationship was observed in mechanically processed samples. This divergence may be explained by age-related alterations in the organizational composition of the ECM, which has been shown to become increasingly stiff and disorganized with advancing age [70,71]. Concurrently, aging ADSCs exhibit an increased expression of senescent markers such as *p16* and *p21*, which are associated with dysfunctional cellular activity and diminished tolerance to physiological stress [72]. As such, enzymatic degradation of the ECM may be less efficient and more heterogeneous in aged tissue, increasing cellular stress and disproportionately affecting fragile or senescent cells upon release. In contrast, mechanical digestion offers a less complete breakdown of the ECM [73]. Consequently, more fragile cells may remain trapped within the residual matrix, obscuring age-related differences in cell viability.

It should be noted that the enzymatic digestion sample size was larger (*n* = 100) and spanned a wider age range (21–88 years), whereas the mechanical digestion samples were fewer (*n* = 14) and limited to older donors (50–88 years). These differences may reduce the ability to detect age-dependent effects in mechanically processed SVF. Nevertheless, the observed trend is supported by prior literature. Cell viability has been shown to decline over a 72 h period post-enzymatic isolation, while viability remained stable in samples that were mechanically processed [41], implying that exposure to proteolytic enzymes may induce additional stress on cells over time. In conjunction with our results, this finding suggests that enzymatic isolation may slightly exacerbate the impact of age on SVF viability. However, enzymatic isolation still displayed higher overall percent viabilities than mechanical digestion, even in elderly donors, indicating that enzymatic digestion is still more reliable in terms of percent viability despite a slight negative association with age.

Interestingly, donor age displayed no significant correlation with TNC for SVF samples that underwent either processing technique. This finding is consistent with previous reports that have also denied a correlation between cell yield and donor age [55,56,74,75]. However, this relationship remains controversial, as other studies have correlated increasing age with diminished SVF yields [63,76]. Variability in isolation protocols and donor demographics may account for this discrepancy. Therefore, further investigation is needed to elucidate the true effect of age on TNC.

### 4.3. Sex

No significant correlation between donor sex and percent viability was observed for either isolation technique. This finding is corroborated by a 2021 study by Andjelkov et al., which similarly reported no relationship between donor sex and percent viability [64]. The lack of a significant correlation between donor sex and percent viability suggests that cell survival is largely dependent on isolation methods and other donor-related factors such as age rather than donor sex. However, additional studies are needed to confirm this finding across larger donor populations.

Female donors exhibited higher TNCs than male donors following enzymatic digestion. This finding was expected and supported by prior literature [62,63,77]. Cremona et al. proposed that this trend may reflect sex-based anatomical differences in the distribution of subcutaneous adipose tissue; larger subcutaneous fat depots in females necessitate more extensive vascularization, which is heavily supported by endothelial progenitor cells [77,78]. Because endothelial progenitor cells also comprise a portion of the SVF [79], increased vascular density may account for the observed association between female sex and higher cell yield. Hormonal influences may further modulate this effect, as estrogen has been shown to upregulate VEGF, contributing to neovascularization and endothelial repair [80,81].

However, this effect was not seen in female donors who underwent mechanical digestion, as no correlation was identified between donor sex and TNC for mechanically isolated SVF samples. Although sample size was limited for mechanical digestion, the absence of a sex-based association with TNC following mechanical digestion suggests that isolation methodology may impact the extent to which intrinsic biological differences are reflected in SVF yield. Unfortunately, very few studies have examined this relationship, as the majority of studies reporting higher TNC in females utilized some form of enzymatic digestion [62,63,77]. As such, further investigation is needed to directly compare isolation techniques across sexes in relation to TNC.

### 4.4. Limitations

Our study was limited by unequal sample sizes between the two isolation methods, reflecting an evolution of clinical procedure and a delayed implementation of quantitative SVF analysis, which may have reduced the ability to draw direct conclusions. Although the post hoc power analysis demonstrated adequate power for detecting differences in percent viability, the comparison of TNC was underpowered, limiting the ability to detect moderate differences in TNC between the two groups.

Additionally, the difference in age range between the two cohorts may have introduced a potential confounding factor. Although the age-adjusted sensitivity analysis supported our findings, these results should be interpreted cautiously, as isolation technique was closely associated with harvesting method. Enzymatic isolations were performed following manual pull harvesting, whereas mechanical isolations were performed following water-assisted harvesting. This variation may confound comparisons attributed to isolation technique alone. As such, future studies with larger, balanced cohorts are needed to fully disentangle these effects. Furthermore, sex distribution was not perfectly balanced across age categories, which may introduce minor confounding when interpreting age-related differences in SVF outcomes.

Lastly, we did not examine the interactions of potential comorbidities such as obesity, smoking status, or cardiovascular disease. We were also unable to determine how isolation technique or donor characteristics may impact the heterogeneity and immunophenotypic composition of the isolated SVF samples, as flow cytometry for CD markers was not routinely performed at the time of SVF collection. Future studies should include phenotypic profiling to better understand how cellular composition varies across isolation methods and donor characteristics, and to assess how subpopulation differences may impact regenerative potential, paracrine signaling, and clinical efficacy.

### 4.5. Future Perspectives

Future studies should include larger, more demographically balanced cohorts to validate our results. Additionally, laboratory tests assessing the growth potential and differentiation capacity of ADSCs present within SVF would be useful to evaluate how variability in SVF yield and viability influences overall clinical efficacy.

## 5. Conclusions

This study highlights the influence of isolation technique and donor characteristics on SVF yield and viability. Enzymatic digestion consistently produced higher cell viabilities compared to mechanical digestion. Isolation technique was not associated with TNC. Increasing donor age was modestly associated with reduced viability following enzymatic digestion, but showed no correlation with viability after mechanical processing. Donor age was not associated with TNC for either isolation method. Donor sex showed no significant association with percent viability for either processing technique. Female donors were associated with higher TNCs following enzymatic digestion. Donor sex was not correlated with TNC following mechanical digestion.

## Figures and Tables

**Table 1:** Summary of patient demographics and corresponding outcome measures as means.

Patient Characteristic		n (%)	Mean Yield (×10^6^) (SD)	Mean Viability (%) (SD)
Age	20–40 years	7 (6.1)	5.39 (4.29)	85.94 (6.04)
	40–60 years	34 (29.8)	7.05 (7.44)	83.01 (15.40)
	60–90 years	73 (64.0)	5.73 (6.09)	81.62 (11.07)
Sex	Female	57 (50.0)	7.53 (5.77)	83.66 (10.22)
	Male	57 (50.0)	4.64 (6.74)	80.96 (13.98)
Isolation technique	Enzymatic	100 (87.7)	6.48 (6.73)	85.80 (7.58)
	Mechanical	14 (12.3)	3.31 (1.68)	57.10 (9.48)

## Data Availability

The original data presented in the study are openly available in FigShare at 10.6084/m9.figshare.31171099.

## Abbreviations

The following abbreviations are used in this manuscript:

SVF	Stromal vascular fraction
ADSC	Adipose-derived stem cell
BMSC	Bone marrow-derived stem cell
TNC	Total nucleated count
MSC	Mesenchymal stem cell
ECM	Extracellular matrix
E2	17-β estradiol
VEGF	Vascular endothelial growth factor
ERα	Estrogen receptors type α
ERβ	Estrogen receptors type β
ATPU	Automatic tissue processing unit
D5LR	5% dextrose in lactated Ringer’s
AO	Acridine orange
DAPI	4′,6-diamidino-2-phenylindole
